# Rapid diagnosis, genome sequencing and culture isolation of a monkeypox virus of clade Ib from a Ugandan during a professional stay in France

**DOI:** 10.1016/j.ijregi.2026.101001

**Published:** 2026-09-02

**Authors:** Stheme de Jubecourt Aymery, Florian Cardona, Gwilherm Penant, Olivier Ferraris, Bernard La Scola, Philippe Colson, Jean-Christophe Lagier

**Affiliations:** 1 Institut Hospitalo-Universitaire Méditerranée Infection, 19-21 boulevard Jean Moulin, 13005 Marseille, France; 2 Assistance Publique-Hôpitaux de Marseille, 264 rue Saint-Pierre, 13005, Marseille, France; 3 CNR Orthopoxvirus, Institut de Recherche Biomédicale des Armées, Unité de Virologie, 1 Place Valérie André, 91220, Brétigny-sur-Orge, France; 4 Aix-Marseille University, Microbes Evolution Phylogeny and Infections (MEPHI), 27 boulevard Jean Moulin, 13005, Marseille, France

**Keywords:** Mpox, Monkeypox virus, Clade Ib, Genome, Uganda, France

## Abstract

A new monkeypox virus (MPXV) lineage, clade Ib, was first identified in September 2023 in South Kivu, Democratic Republic of the Congo. Here, we report the fourth imported case of MPXV clade Ib identified in France and the first publicly available French genome sequence and culture isolate. MPXV clade Ib infection was detected by quantitative polymerase chain reaction (qPCR) and confirmed by a clade Ib-specific qPCR. Whole-genome sequencing using Oxford Nanopore technology and viral culture on Vero E6 cells were both performed in a biosafety level 3 laboratory. Genome classification used Nextclade. Clinical presentation and outcome were without particularities or complications. This case highlights the ongoing international dissemination of MPXV clade Ib and underscores the importance of genomic surveillance and viral isolation for monitoring emerging MPXV lineages in non-endemic countries.

## Highlights

• Imported Ugandan case of monkeypox virus clade Ib in Marseille, France.
• Fourth monkeypox virus clade Ib case reported in France.
• Real-time genome sequencing and culture isolation.
• Clinical presentation without particularities and a favorable, non-severe outcome.

## Introduction

Monkeypox virus (MPXV) was first described in 1959 [1,2] and the first human case was recognized in 1970 [3]. It belongs to the family *Poxviridae* and is an enveloped virus with double-stranded linear DNA approximately 190 kilobase pairs in length that encodes approximately 180 proteins. Two major clades exist: clade I, endemic in Central Africa, and clade II, endemic in Western Africa [2,4]. In non-endemic areas, including Western Europe, only a few sporadic imported cases had been observed until recently [2,4].

A major mpox outbreak occurred in 2022 in many non-endemic countries, mostly in Europe, North America, and Latin America, causing more than 114,000 cases [5,6]. It was unexpectedly sexually transmitted, delineating a new disease, and was due to a new MPXV lineage of clade II named IIb, considered to have emerged in Nigeria [7]. Another MPXV lineage, this time from clade I, was first identified in September 2023 in South Kivu, Democratic Republic of the Congo (DRC) [8]. It was deemed to be genetically related to sequences obtained from samples collected in 2011-2012 in Kivu [9]. Clade Ib has been reported to exhibit lower genetic diversity than clade IIb, while sharing a substantial association with APOBEC3-mediated mutational signatures, as observed for clade IIb. In addition, clade Ib is characterized by a 1,142-bp genomic deletion relative to clade Ia [10]. In sub-Saharan Africa, MPXV clade Ib infections were detected in individuals from both genders and in children, but sexual intercourse involving individuals of both genders was reported to be a major driver of initial and sustained transmission [5,11]. Clade Ib has disseminated to several African countries [11]. In addition, it caused approximately 40 imported cases in non-endemic areas worldwide [11]. Mpox genome sequencing and analyses have been critical in deciphering and monitoring the recent mpox outbreaks in sub-Saharan Africa [12,13]. In France, three MPXV clade Ib cases were reported in 2025 [14], but no genome had been released. Here, we describe a fourth MPXV clade Ib case, diagnosed in Marseille, southeastern France, for which real-time genome sequencing and virus culture allowed us to obtain a viral genome released in international databases and an isolate available for further phenotypic studies.

## Case report and virological investigations

The patient was a man in his 20s from Kampala, Uganda, who traveled to France in 2025 for professional training. Three days after his arrival, he presented with headaches, fever, swollen lymph nodes in the groin area, and skin lesions initially located on the pubic area and the penis, then on the ears, forehead, and all four limbs (Figure 1). The patient did not exhibit any other clinical symptoms. The patient reported having had sexual intercourse with women but reported no contact with anyone infected with MPXV. C-reactive protein was 5.6 mg/l, the white blood cell count was 4.74 × 10^9^/l, creatininemia was 73 µmol/l, liver transaminase levels and the prothrombin index were within usual limits. The patient reported no regular sexual partner and only previous protected sexual intercourse with women.

**Figure 1:**
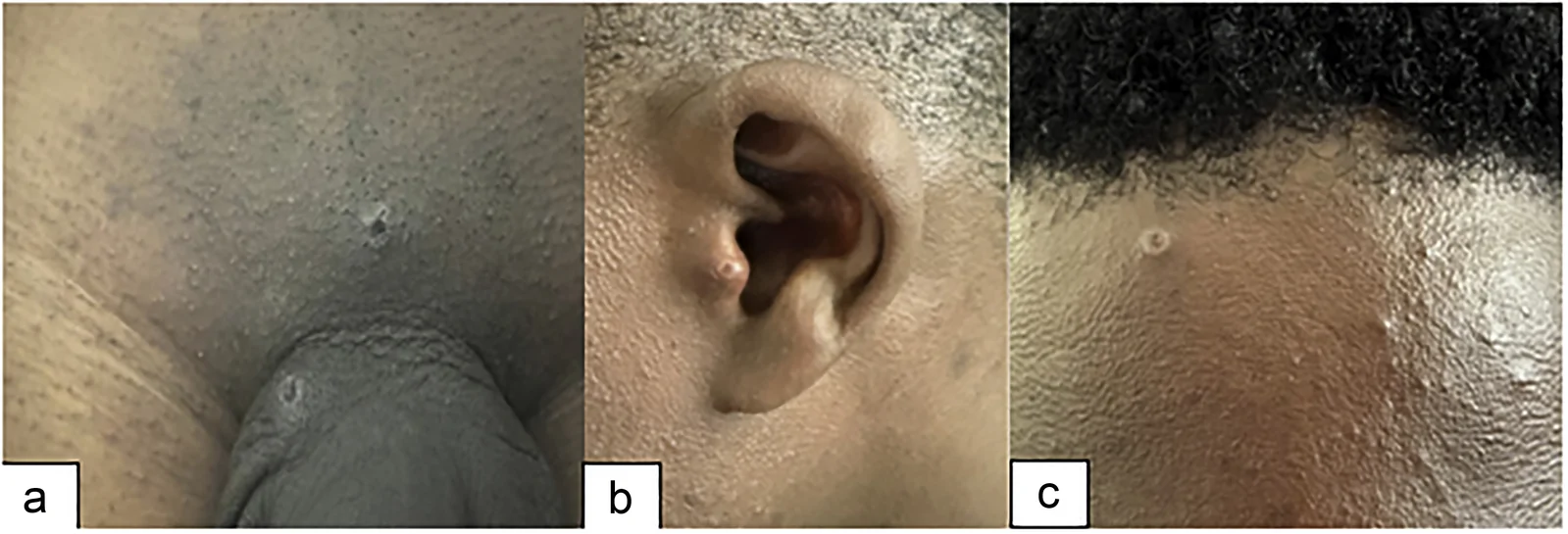
Mpox lesions in the patient. (a) Pubic area; (b) ear lobe; (c) forehead.

Clinical samples were collected at our institute, and virological testing was performed in the biosafety level 3 (BSL3) laboratory. MPVX DNA-specific polymerase chain reaction (PCR) (R-Biopharm, Darmstadt, Germany) performed on an umbilical skin lesion was positive (cycle threshold [Ct] value, 30), and a clade Ib-specific PCR [13] was also performed and was positive. Samples were inoculated the same day on Vero E6 cells, as previously described [15]. A cytopathic effect was observed 5 days post-inoculation, and the viral isolate was produced and then stored at −80°C. In addition, direct real-time next-generation sequencing (NGS) of the MPXV genome was performed the day following culture inoculation in the BSL3 laboratory using Oxford Nanopore technology, as previously reported [15], on a MinION Mk1C instrument (Oxford, UK). This allowed us to assemble, on day 2, a 12,409-base-pair-long genome using EPI2MElabs (Oxford Nanopore) and Minimap2 [16] with reference genome National Center for Biotechnology Information (NCBI) GenBank accession no. OZ254187.1. Subsequent direct NGS performed on a PCR-positive (Ct, 19) skin lesion scab allowed us to obtain a full-length viral genome 2 days later based on 34,166 reads (195,608 nucleotides; Global Initiative on Sharing All Influenza Data (GISAID) [https://gisaid.org/] accession no. EPI_ISL_20349308). Using the Nextclade web application (https://clades.nextstrain.org/), the genome was confirmed to belong to clade Ib, to be most similar to genomes from Germany, the UK, the USA, and Senegal (Figure 2), and to exhibit 129 mutations relative to GenBank genome no. DQ011155.1 (strain Zaire_1979-005). The patient also tested positive for hepatitis B surface antigen and total anti-hepatitis B core antibodies (anti-HBc) with negative immunoglobulin Manti-HBc (Cobas assays, Roche, Meylan, France), and serum hepatitis B virus (HBV) DNA load was 36 IU/mL (Hologic, San Diego, USA), indicating ongoing HBV infection. Serological and PCR testing for human immunodeficiency virus, hepatitis delta virus, hepatitis C virus, and syphilis, and PCR testing for syphilis and other major sexually transmitted bacterial agents were negative. The patient was isolated and treated exclusively with painkillers. Outcome was favorable, with lesions healing and the patient’s discharge from hospital 6 days post-admission without any complications.

**Figure 2:**
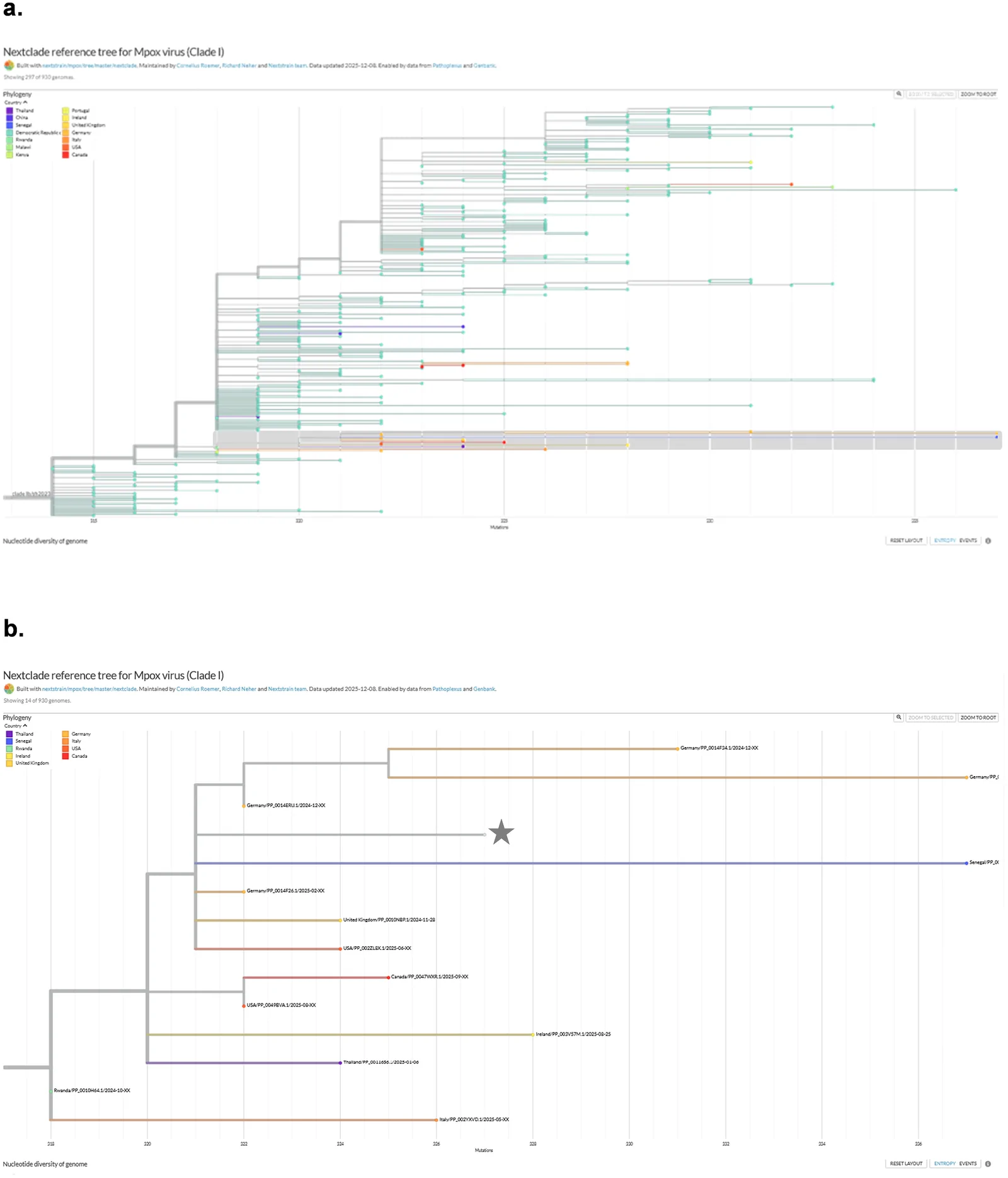
Phylogenies based on whole monkeypox virus genomes. Trees were built with Nextclade [17] and visualized using Auspice.us (https://auspice.us/). Subfigures 1a and 1b show clade Ib genomes at different magnifications, with colors indicating countries. Subfigure 1b shows the part of subfigure 1a that is framed by a gray background. The monkeypox virus genome obtained here is indicated by a gray star in subfigure 1b. Out of a set of 930 genomes as of 21/05/2026.

## Discussion

Here, we obtained an MPXV clade Ib genome from France released in a public sequence database and a culture isolate usable for further phenotypic and experimental studies. Both the genome and the isolate were shared with the French Reference Center-Expert Laboratory for orthopoxvirus. Considerable efforts have been put into sequencing and analysis of the genomes of MPXV since the 2022 outbreak in non-endemic areas [12]. In contrast, culture isolation of this virus has been rarer due to the use of sequencing at the forefront of virus characterization. Thus, the feasibility of genome NGS increased considerably during the severe acute respiratory syndrome coronavirus 2 (SARS-CoV-2) pandemic, whereas viral culturing is still performed only in highly specialized centers because it requires a BSL3 laboratory, as is the case for reference healthcare facilities for epidemic and biological risk in France. As a matter of fact, the genome obtained here from the culture isolate is the first clade Ib genome available from France to date, while 218 of the 229 clade IIb MPXV genomes available from France have also been obtained from our institute (Gisaid (https://gisaid.org/) Accession no. EPI_ISL_14863048, EPI_ISL_14863050-EPI_ISL_14863066, EPI_ISL_19643020, EPI_ISL_20361066-EPI_ISL_20361201, EPI_ISL_20361203-EPI_ISL_20361213, EPI_ISL_20361215-EPI_ISL_20361226, EPI_ISL_20361228-EPI_ISL_20361267), and they include 198 genomes that were obtained from positive culture supernatants [15]. Thus, genomic surveillance of MPXV is not exhaustive at this country scale despite having largely proved to be highly valuable with SARS-CoV-2 for the characterization of virological and epidemiological features and for the study of immune escape and viral resistance.

The present case illustrates the emerging circulation of MPXV clade Ib outside endemic African regions. The first mpox case of clade Ib outside Africa was reported in a Swedish traveler returning in August 2024 from an area in Central Africa [18]. Thereafter, for the time period from August 2024 through July 2025, Laurenson-Schafer et al. [19] reported 89 travel-related MPXV clade Ib infections diagnosed in 33 countries. These cases mostly involved men, and approximately one third led to secondary transmission, the risk of which was highest among sexual contacts, followed by household contacts. The present case was the fourth reported case of clade Ib in France by the end of 2025, all of which were linked to international travel [20]. Regarding the three previous cases, the first one was reported in December 2024 in a patient who had been in contact with individuals who had traveled to an African country where MPXV clade Ib was circulating. The second and third cases were confirmed in February and March 2025 and were imported from two such countries. In the present case, infection originated in Uganda, where clade Ib is circulating [20]. So far, more than 8000 MPXV clade Ib cases have been reported in Uganda since July 2024, making this country the second most affected country after DRC [20]. Unlike what was seen in Ireland, our case did not cause an epidemic, as the patient did not report sexual intercourse during his stay in France and was hospitalized for 6 days [21]. MPXV clade I was primarily reported as associated with a higher fatality rate than clade II [5]. In fact, the fatality rate attributed to clade IIb was <1% in South Kivu and Burundi and was 0.4% according to a report by the World Health Organization [22], [23]. Here, clinical presentation and outcome were typical and non-severe, in congruence with previously reported clade Ib cases in non-endemic areas [5]. The patient was also diagnosed with HBV infection, most probably chronic. This did not necessarily suggest sexual transmission of this virus, since, for instance, HBV vaccination of infants and a birth dose of HBV vaccine were only introduced in Uganda in 2022 and 2025, respectively.

MPXV of clade Ib displays interesting genetic features. Intra-clade Ib diversity appears lower so far than that for clade IIb [10], which raises the question of whether both viruses have the same transmission efficiency and replicative capabilities. However, APOBEC3-mediated signature mutations seem to occur at a similar rate in clade Ib and clade Ia and at a greater rate in these clades than in those circulating before 2022 [10]. This was deemed to suggest sustained human-to-human transmission [10]. A particular feature of clade Ib compared with clade Ia and shared with clade II is the existence of a 1142-bp-long deletion located at positions ≈20.3-21.5 kb relative to GenBank genome number. NC_063383.1, which corresponds to the OPG032-OPG033 region. This large deletion is hence associated with the loss of the OPG032 gene that is homologous to the vaccinia virus complement control protein-encoding gene (C4L) [10]. This gene was reported to disrupt the host complement cascade [24], and attempts were also made to link it to a lower fatality rate, but previous studies conducted in non-human animals reported conflicting results with either lower or the same disease severity and differences in adaptive immune responses in the presence or absence of this gene [10]. Thus, the impact of this gene on virus pathogenicity remains unclear. Notwithstanding, gene losses were frequent among orthopoxviruses, predominant in speciation events, and commonly impacted host range [25], and at least one was associated with evolutionary benefit rather than disadvantage [6]. In fact, genes of such zoonotic viruses as orthopoxviruses may become useless in humans after switching to a new host, and this was also observed in other viruses, including SARS-CoV-2 and other human coronaviruses [26].

Overall, this case highlights the importance of clinical vigilance and epidemiological surveillance in response to the emergence of new MPXV lineages, even in non-endemic areas. The early detection and isolation of cases can limit the spread of the virus, and tracking and characterizing imported infections can improve our understanding of the clinical and virological characteristics of new lineages. Real-time NGS and culture are highly valuable tools with respect to the information that can be obtained from viral genomes and culture isolates, respectively. The very recent emergence of several different clade Ib/IIb recombinant MPXV [27] still reinforces the interest of such surveillance and study tools.

## Funding

This work was supported by the French Government under the “Investments for the Future” program managed by the National Agency for Research (ANR), Méditerranée-Infection 10-IAHU-03, by the Contrat Plan Etat-Région, and by the European funding FEDER IHUPERF.

## Ethical approval

Access to the patient’s biological and registry data from the hospital information system was approved by the data protection committee of Assistance Publique-Hôpitaux de Marseille and was recorded in the European General Data Protection Regulation registry under number APHM 25-366; this study was approved by our institution’s scientific and ethics committee under no. 25-379. In addition, the patient signed a consent form authorizing the consultation and publication of data from his medical records.

## CRediT authorship contribution statement

**Stheme de Jubecourt Aymery:** Writing – review & editing, Writing – original draft, Validation, Data curation. **Florian Cardona:** Writing – review & editing, Validation, Data curation. **Gwilherm Penant:** Writing – review & editing, Validation, Investigation. **Olivier Ferraris:** Writing – review & editing, Validation. **Bernard La Scola:** Writing – review & editing, Validation, Funding acquisition. **Philippe Colson:** Writing – review & editing, Writing – original draft, Validation, Supervision, Formal analysis, Data curation, Conceptualization. **Jean-Christophe Lagier:** Writing – review & editing, Validation, Supervision, Formal analysis, Conceptualization.

## Declaration of competing interest

PC and BLS are scientific advisors to BioSellal and Triber Global. The other authors have no competing interests to declare. Funding sources had no role in the design and conduct of the study; collection, management, analysis, and interpretation of the data; and preparation, review, or approval of the manuscript.

## References

[1] Magnus P.V., Andersen E.K., Petersen K.B., Birch-Andersen A. (1959). A pox-like disease in cynomolgus monkeys. Acta Pathol Microbiol Scand.

[2] Alakunle E., Kolawole D., Diaz-Cánova D., Alele F., Adegboye O., Moens U. (2024). A comprehensive review of monkeypox virus and mpox characteristics. Front Cell Infect Microbiol.

[3] Foster S.O., Brink E.W., Hutchins D.L., Pifer J.M., Lourie B., Moser C.R. (1972). Human monkeypox. Bull World Health Organ.

[4] Effiong F.B., Elebesunu E.E., Ogunniyi T.J., Olawuyi D.A., Ekpor E., Ahiadorme M. (2025). Mpox surveillance in endemic regions: a scoping review of trends, challenges, and recommendations. BMC Infect Dis.

[5] Srivastava S., Sharma D., Sridhar S.B., Kumar S., Rao GSNK, Budha R.R. (2025). Comparative analysis of Mpox clades: epidemiology, transmission dynamics, and detection strategies. BMC Infect Dis.

[6] Brinkmann A., Kohl C., Pape K., Bourquain D., Thürmer A., Michel J. (2023). Extensive ITR expansion of the 2022 Mpox virus genome through gene duplication and gene loss. Virus Genes.

[7] Mitjà O., Ogoina D., Titanji B.K., Galvan C., Muyembe J-J, Marks M. (2023). Monkeypox. Lancet.

[8] Masirika L.M., Udahemuka J.C., Schuele L., Nieuwenhuijse D.F., Ndishimye P., Boter M. (2025). Epidemiological and genomic evolution of the ongoing outbreak of clade Ib mpox virus in the eastern Democratic Republic of the Congo. Nat Med.

[9] McCollum A.M., Nakazawa Y., Ndongala G.M., Pukuta E., Karhemere S., Lushima R.S. (2015). Human monkeypox in the Kivus, a conflict region of the Democratic Republic of the Congo. Am J Trop Med Hyg.

[10] Satheshkumar P.S., Gigante C.M., Mbala-Kingebeni P., Nakazawa Y., Anderson M., Balinandi S. (2025). Emergence of clade Ib monkeypox virus-current state of evidence. Emerg Infect Dis.

[11] Pareek A., Singhal R., Pareek A., Chuturgoon A., Apostolopoulos V., Chattu V.K. (2025). Global spread of clade Ib mpox: a growing concern. Lancet Microbe.

[12] Desingu P.A., Rubeni T.P., Nagarajan K., Sundaresan N.R. (2024). Molecular evolution of 2022 multi-country outbreak-causing monkeypox virus Clade IIb. iScience.

[13] Schuele L., Masirika L.M., Udahemuka J.C., Siangoli F.B., Mbiribindi J.B., Ndishimye P. (2024). Real-time PCR assay to detect the novel Clade Ib monkeypox virus, September 2023 to May 2024. Euro Surveill.

[14] World Health Organization European Region (2025).

[15] Colson, Philippe, Le Targa L, Valade M, Beye M, Delerce J, et al. Analyse de 217 génomes du virus monkeypox (Mpox) obtenus par séquençage de nouvelle génération directement à partir d’échantillons cliniques ou après culture dans le sud-est de la France lors de l’épidémie de 2022. Journées francophones de virologie 2024.

[16] Li H. (2018). Minimap2: pairwise alignment for nucleotide sequences. Bioinformatics.

[17] Aksamentov I., Roemer C., Hodcroft E., Neher R. (2021). Nextclade: clade assignment, mutation calling and quality control for viral genomes. JOSS.

[18] Treutiger C-J, Filén F., Rehn M., Aarum J., Jacks A., Gisslén M. (2024). First case of mpox with monkeypox virus clade Ib outside Africa in a returning traveller, Sweden, August 2024: public health measures. Euro Surveill.

[19] Laurenson-Schafer H., McMenamin M., Ebbarnezh L.K., Karagiannis I., Biaukula V., Lewis R. (2026). Characteristics and transmission dynamics of global travel-related mpox cases caused by clade Ib monkeypox virus. Emerg Infect Dis.

[20] World Health Organization (2025).

[21] McLoughlin M., Fahey L., Carr M., Caceda B., Igoe D., Dean J. (2025). Nosocomial transmission in a monkeypox virus clade Ib outbreak, Ireland, August to October 2025. Euro Surveill.

[22] WHO Health Organization (2026).

[23] Du M., Liu M., Niu B., Liu J. (2024). The global alarm bell is ringing due to the threat of potential severe cases and deaths caused by clade I of monkeypox virus. Lancet Infect Dis.

[24] Liszewski M.K., Leung M.K., Hauhart R., Buller R.M.L., Bertram P., Wang X. (2006). Structure and regulatory profile of the monkeypox inhibitor of complement: comparison to homologs in vaccinia and variola and evidence for dimer formation. J Immunol.

[25] Senkevich T.G., Yutin N., Wolf Y.I., Koonin E.V., Moss B. (2021). Ancient gene capture and recent gene loss shape the evolution of Orthopoxvirus-host interaction genes. mBio.

[26] Colson P., Delerce J., Fantini J., Pontarotti P., La Scola B., Raoult D. (2023). The return of the “Mistigri” (virus adaptative gain by gene loss) through the SARS-CoV-2 XBB.1.5 chimera that predominated in 2023. J Med Virol.

[27] Pullan I Everall, Rebecca Doherty, Lucy Crossman, Emma Wise, Hassan Hartman, et al. Inter-Clade Recombinant Mpox Virus Detected in England in a Traveller Recently Returned from Asia. 2025.

